# Isatuximab monotherapy in patients with refractory T‐acute lymphoblastic leukemia or T‐lymphoblastic lymphoma: Phase 2 study

**DOI:** 10.1002/cam4.4478

**Published:** 2022-02-02

**Authors:** Nicolas Boissel, Patrice Chevallier, Vadim Doronin, Laimonas Griskevicius, Alexey Maschan, James McCloskey, Alessandro Rambaldi, Giuseppe Rossi, Andrey Sokolov, Ulla Wartiovaara‐Kautto, Corina Oprea, Giovanni Abbadessa, Alice Gosselin, Sandrine Macé, Xavier Thomas

**Affiliations:** ^1^ Department of Adult Hematology Saint‐Louis Hospital Institut de Recherche Saint‐Louis University of Paris Paris France; ^2^ CHU Hôtel‐Dieu Service d'Hématologie Clinique Nantes France; ^3^ Municipal Clinical Hospital Moscow Russian Federation; ^4^ Hematology, Oncology and Transfusion Medicine Center Vilnius University Hospital Santaros Klinikos Vilnius University Vilnius Lithuania; ^5^ Federal Research Center for Pediatric Hematology, Oncology, and Immunology Moscow Russian Federation; ^6^ Division of Leukemia John Theurer Cancer Center at Hackensack University Medical Center Hackensack New Jersey USA; ^7^ Università Statale di Milano, Milan and Azienda Socio Sanitaria Territoriale Bergamo Italy; ^8^ Divisione di Ematologia ASST Spedali Civili Brescia Italy; ^9^ National Research Center for Hematology of the Russian Ministry of Health Moscow Russian Federation; ^10^ Comprehensive Cancer Center Department of Hematology Helsinki University Hospital Helsinki Finland; ^11^ Sanofi Vitry‐sur‐Seine France; ^12^ Sanofi Cambridge Massachusetts USA; ^13^ Hematology Department Hospices Civils de Lyon Lyon‐Sud University Hospital Pierre Bénite France

**Keywords:** acute lymphoblastic leukemia, isatuximab, monoclonal antibodies, monotherapy, T lymphoblastic lymphoma

## Abstract

The poor prognosis of acute T‐cell lymphoblastic leukemia (T‐ALL) and T‐cell lymphoblastic lymphoma (T‐LBL) in older adults and patients with relapsed/refractory illness is an unmet clinical need, as there is no defined standard of care and there are few treatment options. Abnormally elevated CD38 expression in T‐ALL and T‐LBL is associated with tumor expansion and disease development, making CD38 a potential target for anti‐T‐ALL and T‐LBL treatment. Isatuximab is a monoclonal antibody that binds to a specific epitope on CD38. The purpose of the study was to assess the efficacy and safety of isatuximab monotherapy in a phase 2, multicenter, one‐arm, open‐label study in patients with relapsed or refractory T‐ALL or T‐LBL (Clinical Trials.gov identifier NCT02999633). The primary endpoint was to assess the efficacy of isatuximab by overall response rate (ORR). An interim analysis based on the efficacy and safety of isatuximab in the first 19 patients enrolled was scheduled, however only 14 patients were enrolled in the study. No patient achieved complete response (CR) or CR with incomplete peripheral recovery. Most patients (11 [78.6%]) developed progressive disease and had progressive disease as their best response. A total of 10 (71.4%) patients had treatment emergent adverse events considered treatment‐related, with infusion reactions as the most frequent drug‐related TEAE, occurring in 8 (57.1%) patients. Despite the low efficacy of isatuximab in the current study, it is likely that the use of immunotherapy medication in T‐ALL will be expanded through logically targeted approaches, together with advances in the design of T‐cell therapy and clinical experience and will provide restorative options beyond chemotherapy and targeted treatments.

## 1 INTRODUCTION

Adult acute T‐cell lymphoblastic leukemia (T‐ALL) and T‐lymphoblastic lymphoma (T‐LBL) are clonal illnesses that affect bone marrow progenitor cells.^1^ Approximately 40%–50% of adult patients with T‐ALL or T‐LBL on treatment will relapse and overall survival in adults over the age of 50 years is poor, with less than 25% of patients who had relapsed still alive five years after diagnosis.^2^, ^3^, ^4^ The poor prognosis in older adults and in patients with relapsed/refractory disease is an unmet clinical problem, as there is no defined standard of care and there are few treatment options. Consequently, targeted compounds are required to improve antileukemic activity and reduce the need for treatment intensification with higher‐dose chemotherapy, which is poorly tolerated, especially in older patients.^5^

Abnormally elevated CD38 expression in T‐ALL and T‐LBL is associated with tumor expansion and progressive disease, making CD38 a potential target for anti‐T‐ALL and T‐LBL treatment.^6^, ^7^ Isatuximab is a monoclonal antibody that binds to a specific epitope on CD38. Isatuximab's modes of action include immediate tumor cell lysis through antibody‐dependent cellular cytotoxicity (ADCC), antibody‐dependent cellular phagocytosis (ADCP), complement‐dependent cytotoxicity, and direct apoptosis. Isatuximab modulates the activity of immune effector cells, including natural killer (NK) cells, through the binding of the CD38 enzyme. Isatuximab also elicits longer‐term immunomodulation, which includes an increase in CD3+ T‐cells, a decrease in T regulatory cells, and the induction of myeloma specific antitumor immunity.^8^, ^9^, ^10^, ^11^, ^12^, ^13^ Isatuximab (Sarclisa^®^) is approved in a number of countries in combination with pomalidomide and dexamethasone for the treatment of adult patients with relapsed/refractory multiple myeloma who have received at least two prior therapies, including lenalidomide and a proteasome inhibitor. Isatuximab in combination with carfilzomib and dexamethasone is approved in the United States, for the treatment of adult patients with relapsed or refractory multiple myeloma who have received one to three prior lines of therapy, and in the European Union, for the treatment of adult patients with relapsed multiple myeloma who have received at least one prior therapy.

A preclinical study showed that isatuximab has significant *in vitro* and *in vivo* activity against ALL cells with a robust ADCC and ADCP effect that is clearly correlated with CD38 expression levels in both T‐ALL and B cell‐acute lymphoblastic leukemia (B‐ALL)^14^ The purpose of the study was to evaluate the efficacy and safety of isatuximab monotherapy in patients with relapsed or refractory T‐ALL or T‐LBL.

## 2 MATERIALS AND METHODS

This was phase 2, single‐arm, multicenter, multinational, open‐label study evaluating the efficacy and safety of isatuximab in patients with relapsed or refractory T‐ALL/T‐LBL (ClinicalTrials.gov identifier NCT02999633). The study was carried out in six countries (Finland, France, Italy, Lithuania, the Russian Federation, and the United States) in two stages using a Simon's optimal two‐stage design. There was an interim analysis after stage 1 based on efficacy and safety of isatuximab in the first 19 patients (note that only 14 patients were enrolled) and the study was to proceed to stage 2 if >3/19 patients showed a clinical response to treatment.

The protocol was approved by independent ethics committees and institutional review boards at all participating institutions before the study commenced. Written informed consent was obtained from all participants prior to inclusion in the study. The study was conducted in accordance with the Declaration of Helsinki and the International Conference on Harmonization Guidelines for Good Clinical Practice.

Participants were eligible if they were >16 years at the time of signing the consent form and had a confirmed diagnosis of relapsed ALL (T‐ or B‐cell origin) including lymphoblastic lymphoma or relapsed AML. Participants must have been previously treated for their disease and have relapsed or were refractory to their most recent treatment. Participants were excluded if there was evidence of an ongoing infection or seropositivity to human immunodeficiency virus, had uncontrolled or active hepatitis B or hepatitis C infections; participants had a second malignancy other than basal cell or squamous cell carcinoma; had cardiomyopathy; a history of thrombophilic disease; Eastern Cooperative Oncology Group performance status >2 or Lansky score <70; had a total bilirubin >2.5 times the upper limit of normal (ULN); aminotransferase or alkaline phosphatase levels >5 times ULN; serum creatinine levels >2 times ULN; had any serious comorbid condition that could have interfered with the safety of the study intervention or affected compliance to the study medication.

Study medications included: (i) ALL cohorts: 20 mg/kg of isatuximab infused weekly (this could be modified based on modeling and PK assessment on the first 20 participants) Days 1, 8, 15, 22, 29, 43, and 57; (ii) AML cohort: 20 mg/kg of isatuximab infused weekly (this could be modified based on modeling and PK assessment on the first 20 participants) Days 1, 8, and 15 (mandatory for Cycles 1 and 2). Cytarabine administered to the AML cohort only: 60 mg/m^2^, mandatory for Cycle 1 Day 8, optional for Cycle 1 Days 10 and 12, and optional for Cycle 2. Dexamethasone ALL cohorts: Dexamethasone 10 mg/m^2^ (maximum 20 mg) intravenous (IV) or oral (PO) administration on Days ‐3, ‐2, ‐1 before isatuximab administration, 1, 8, 15 to 19, 22, and 29 to 33 for the induction period and Days 43 to 47, and 57 during the consolidation period. AML cohort: Dexamethasone 10 mg/m^2^ (maximum 20 mg) IV or PO optional on Days ‐3, ‐2, ‐1; premedication before isatuximab administration, on Days 1, 8, and 15 during the induction period (mandatory at Cycle 1 and before first isatuximab infusion at Cycle 2). Doxorubicin ALL cohorts only: 10 mg/m^2^ Days 8 and 9 of the induction period. Vincristine ALL cohorts only: 1.5 mg/m^2^ on Days 10, 17, 24, and 31 during the induction period (should not exceed 2 mg per infusion in any patient); Day 38 during the consolidation period. Cyclophosphamide ALL cohorts only: 440 mg/m^2^ on Days 50 to 54 inclusive, during the consolidation period.

If clinical signs of an infusion‐related reaction Grade 1 developed, isatuximab infusion interruption or intervention was not indicated. For Grade 2 infusion reactions, isatuximab therapy or infusion interruption was indicated, but if the patient responded promptly to symptomatic treatment (e.g., antihistamines, non‐steroid anti‐inflammatory drugs, narcotics, or IV fluids). In Grade 2 infusion reactions, additional premedication with IV diphenhydramine 25 mg IV (or equivalent) and/ or IV methylprednisolone 100 mg (or equivalent) was administered as needed. Isatuximab was resumed only after patient recovery, with slower infusion rate and with close monitoring. If severe or life‐threatening (Grade 3 or 4) infusion reactions occurred, isatuximab infusion was stopped and additional premedication with diphenhydramine 25 mg IV (or equivalent) and/ or IV methylprednisolone 100 mg (or equivalent) and/or epinephrine was administered as required.

The primary endpoint was to assess the efficacy of isatuximab by overall response rate (ORR). Secondary endpoints included duration of response,^15^ progression‐free survival, immunogenicity of isatuximab in patients with T‐ALL/T‐LBL, minimal residual disease, and the safety profile of isatuximab. Overall,^16^ patients were screened but two patients failed screening (one patient had an ongoing unspecified infection and one patient did not have relapsed or refractory T‐ALL/T‐LBL).

Sample size calculations were based on a 2‐stage Simon’s minimum‐maximum design in 3 cohorts (T‐ALL, B‐ALL, and AML). In the T‐ALL cohort, if a maximum of 24 evaluable participants were be enrolled, this sample size would provide 80% power to reject the null hypothesis that the complete response (CR + CRi) rate is ≤60% if the CR rate is ≥80%, based on a 1‐sided exact binomial test at a significance level of 0.1. At Stage 1, a total of 11 evaluable participants would be enrolled and proceed to Stage 2 if more than 6 responses were observed.

## 3 RESULTS

The demographic and baseline characteristics of the 14 enrolled patients are shown in Table 1. The median age was 33.0 years (range 16.0–74.0 years), with three (21.4%) patients aged between 65 and 75 years. The majority of patients (*n* = 12, 85.7%) were male. At baseline, all 14 patients (100%) had an Eastern Cooperative Oncology Group (ECOG) performance status score of ≤2, with 11 (78.6%) patients having an ECOG score of <2. At initial diagnosis, most patients had T‐ALL (11 patients, 78.6%), with the remaining patients having T‐LBL. Among the 14 patients enrolled in the study, all had been given at least one prior leukemia/lymphoma treatment regimen and 5 (35.7%) patients had been given ≥8 prior lines of treatment before the start of the study. A total of eight (57.1%) patients had received at least one prior allogeneic transplant and six (42.9%) patients underwent prior radiotherapy treatment for leukemia/lymphoma.

**TABLE 1:** Summary of disease characteristics at baseline

	Isatuximab 20 mg/kg (*N* = 14)
Age, years	
Median (minimum: maximum)	33.0 (16.0: 74.0)
Age group, years, *n* (%)	
<65	11 (78.6)
65–75	3 (21.4)
Gender, *n* (%)	
Female	2 (14.3)
Male	12 (85.7)
ECOG performance status *n* (%)	
0	2 (14.3)
1	9 (64.3)
2	3 (21.4)
Initial diagnosis, *n* (%)	
T‐acute lymphoblastic leukemia	11 (78.6)
T‐lymphoblastic lymphoma	3 (21.4)
Time from initial diagnosis of leukemia/lymphoma to first dose administered	
Median, years (minimum: maximum)	1.39 (0.4: 6.8)
At least one previous allogeneic stem cell transplant, *n* (%)	8 (57.1)
Disease status at study entry, *n* (%)	
Relapsed only	7 (50.0)
Refractory only	5 (35.7)
Relapsed and refractory	2 (14.3)
Number of prior lines of treatment	
Median (minimum: maximum)	5.50 (2.0: 12.0)
Number of prior lines of treatment, *n* (%)	
2	2 (14.3)
3	2 (14.3)
4	2 (14.3)
5	1 (7.1)
6	1 (7.1)
7	1 (7.1)
≥8	5 (35.7)
Number of prior salvage therapy regimens	
Number of patients	6
Median (minimum: maximum)	2.5 (1.0: 3.0)

Abbreviation: ECOG, Eastern Cooperative Oncology Group.

The ORR is summarized in Table 2. No patient achieved complete response (CR) or CR with incomplete peripheral recovery.^16^ Most patients developed progressive disease (11 [78.6%] patients) and had progressive disease as their best response. Global response data were not available for three patients who died of aplasia without a response evaluation. One patient who withdrew from the study because of suspected progressive disease began his post‐treatment cancer therapy one day before confirmation of disease progression. Therefore, this patient was considered not evaluable and was excluded from the efficacy response data. No patient had a response in blood, bone marrow, extramedullary lesions, or central nervous system. Within 2 weeks of starting isatuximab therapy, the disease progressed in 4/14 (28.6%) patients and, as isatuximab treatment requires 4 weeks to reach optimal therapeutic levels, those patients did not have sufficient time to optimally respond to isatuximab therapy.

**TABLE 2:** Summary of overall response rate

	Isatuximab 20 mg/kg (*N* = 14)
Complete response (CR), *n*	0
Complete response with incomplete peripheral recovery (CRi),^16^ *n*	0
Relapsed disease, *n*	0
Progressive disease, *n* (%)	12 (87.5%)^a^
Responders, CR or CRi, *n* (95% CI)	0 (0.0%–23.2%)

^a^ 11 patients had progressive disease as best response (one patient, who withdrew from the study because of suspected progressive disease, had his post‐treatment cancer therapy started 1 day before the confirmation of disease progression).

CD38 expression (relative density of CD38 and relative occupancy of CD38 on the surface of peripheral blood and bone marrow blast cells) was detected at baseline in 10 of 14 patients (Table 3). The median percentage of cells expressing CD38 was 87.90% (range 20.9%–98.4%) in peripheral blood and 90.95% (range 12.2%–98.6%) in bone marrow aspirates. The relative density (RD) of CD38 as assessed by specific antibody binding capacity (sABC) was available for 10 of 14 patients treated with isatuximab 20 mg/kg. The median RD value was 11,093.50 (range 181.0–76,131.0) sABC/cell in blood and 6817.50 (range 108.0–44,872.0) sABC/cell in bone marrow aspirates. CD38 receptor occupancy analysis showed that isatuximab was able to bind to blast cells before and after treatment (Table 3).

**TABLE 3:** Summary of CD38 receptor occupancy/receptor density

	Isatuximab 20 mg/kg (*N* = 14)	
	White blood cells	Bone marrow
CD38 receptor density of cancer cells at baseline (sABC/cell)^a^		
Number of patients	10	10
Median	11,093.50	68,17.50
Minimum: Maximum	181.0: 76,131.0	108.0: 44,872.0
CD38 positivity at baseline (%)		
Number of patients	10	10
Median	87.50	90.95
Minimum: Maximum	20.9: 98.4	12.2: 98.6
CD38 receptor occupancy of cancer cells at baseline (%)		
Number of patients	10	10
Median	61.60	52.31
Minimum: Maximum	24.0: 84.8	29.4: 73.9
CD38 occupancy of cancer cells on treatment (%)		
Number of patients	4	3
Median	57.96	63.99
Minimum: Maximum	26.6: 75.4	61.7: 73.0

Abbreviation: sABC, surface antibody binding capacity.

^a^ CD38 receptor density and receptor occupancy data at baseline were available for ten out of 14 patients in the study.

Only 4 (28.6%) treated patients started their second cycle of isatuximab and the median cumulative dose was 70.19 mg/kg. The median duration of exposure was 3.5 weeks (range 1.0–12.1 weeks).

Treatment‐emergent adverse events (TEAEs) are shown in Table 4. All 14 (100%) patients had at least one TEAE, with 12 (85.7%) patients having at least one TEAE of grade ≥3 intensity. The standard organ class disorders most commonly reported in patients in the study were injury, poisoning, procedural complications and general disorders, and administration site conditions, with eight (57.1%) patients in each.

**TABLE 4:** Summary of drug‐related treatment‐emergent adverse events (all grades and ≥3): Safety population

Primary system organ class Preferred term, *n* (%)	Isatuximab 20 mg/kg (*N* = 14) Drug‐related	
	All grades	Grade ≥ 3
Any event	10 (71.4)	3 (21.4)
Infections and infestations	1 (7.1)	1 (7.1)
Bronchiolitis	1 (7.1)	0
Pneumonia	1 (7.1)	1 (7.1)
Blood and lymphatic system disorders	1 (7.1)	1 (7.1)
Neutropenia	1 (7.1)	1 (7.1)
Thrombocytopenia	1 (7.1)	1 (7.1)
Immune system disorders	1 (7.1)	0
Cytokine release syndrome	1 (7.1)	0
Gastrointestinal disorders	1 (7.1)	1 (7.1)
Pancreatitis	1 (7.1)	1 (7.1)
Renal and urinary disorders	1 (7.1)	0
Urinary retention	1 (7.1)	0
Injury, poisoning and procedural complications	8 (57.1)	0
Infusion reactions	8 (57.1)	0

A total of ten (71.4%) patients had TEAEs regarded by the investigator to be related to treatment, with infusion reactions as the most frequent drug‐related TEAE, occurring in eight (57.1%) patients. All infusion reactions transpired during first infusion and were mild (grade 1 or 2) in intensity. Overall, 11 patients died during the study, six patients died within 30 days from the last dose of treatment (five patients due to disease progression and one patient due to acute myocardial infarction not considered related to study treatment). Of the five patients who died more than 30 days from the last dose, four patients died due to disease progression and one patient died for a reason listed as “other”.

## 4 DISCUSSION

Natural killer cells are important in the surveillance and cytotoxic eradication of malignant leukemic cells.^17^ The stimulation of NK cells to eliminate tumor blasts in ALL may be crucial to achieve a favorable outcome of therapy and long‐term remission of the patients. Isatuximab promotes NK cell‐mediated ADCC and ADCP in tumor cells in MM by crosslinking CD38 and CD16.^18^ Furthermore, preclinical studies have shown that isatuximab has significant activity against ALL cell lines with a robust ADCC and ADCP effect, with a clear association between the expression of CD38 and isatuximab activity in both T‐ALL and B‐ALL^14^

CD38 is uniformly manifested across leukemic blasts of patients with T‐ALL at all stages of the disease.^19^ Furthermore, cytotoxic chemotherapy for the treatment of ALL does not reduce the manifestation of CD38 at numerous timepoints during the course of treatment. Even though there is a high expression of CD38 in T‐ALL, the expression is not as high as observed in MM,^14^, ^18^ which may have contributed to the poor outcome observed in the current study.^20^ However, the threshold for isatuximab activation of NK‐cell ADCC is lower in T‐ALL and B‐ALL than that observed in MM.^14^, ^18^ Natural killer cell numbers were not assessed in the peripheral blood nor did we assess NK allotypes. Therefore, it is possible that the highly pretreated patient cohort in our study may have experienced significant immunosuppression in preceding therapies. Furthermore, it is likely that some of the patients in our study may have had the F/F allotype which does not facilitate binding to NK for ADCP and this may have been a mitigating factor for the poor response to isatuximab monotherapy in the current study. Given the uniform nature of the expression of CD38 in T‐ALL and T‐LBL patients, it is feasible that isatuximab could be efficacious in patients with less advanced disease or if used in conjunction with chemotherapy or other targeted therapies, as is the case when isatuximab is used in conjunction with pomalidomide and dexamethasone in MM. Differences in blast cell kinetics and disease burden between MM and ALL may also account for the lack of efficacy of isatuximab monotherapy in relapsed or refractory ALL compared with MM.^15^ A further consideration is that the median duration of isatuximab therapy in the study was 3.5 weeks, which may not have been sufficient time to achieve an optimal response. Also, CD38 expression should have been tested on lymphoma tissue as well as bone marrow.

Despite the low efficacy of isatuximab in the current study, it is likely that the use of immunotherapy treatments in ALL will be expanded through targeted approaches, alongside advances in chimeric antigen receptor T‐cell therapy design and clinical experience and will provide medicinal options beyond chemotherapy and targeted treatments.^21^ Also, despite the absence of evidence for the efficacy of rituximab, as a single agent in anti‐CD20 Philadelphia‐negative B‐ALL treatment, the addition of rituximab to chemotherapy demonstrated a reduction in the incidence of relapsed or refractory B‐ALL.^20^

In conclusion, the major limitation of the study was that it included heavily pretreated patients that had received multiple previous lines of therapy. Overall, 14 patients were treated but the study was stopped due to lack of efficacy of isatuximab in adult patients with relapsed/refractory T‐ALL or T‐LBL. Most patients (*n* = 12, 85.7%) had progressive disease during the treatment period, making it unlikely to achieve >3/19 responses required to continue the study. Two (14.3%) patients discontinued the study due to adverse events. Most patients, eight (57.1%) patients had mild infusion reactions and one (7.1%) patient developed a cytokine release syndrome. T‐ALL is an aggressive disease and the expression of CD38 is not sufficient to allow a biologic agent to be effective as monotherapy in heavily pretreated patients. Combination therapy with chemotherapy may be more effective in patients who have not received previous rounds of intensive treatment. Most patients in the study discontinued treatment before optimal blood levels of isatuximab could be reached. The poor response and safety profile of isatuximab monotherapy in this study led to an unsatisfactory risk/benefit ratio for this cohort of patients.

## CONFLICTS OF INTEREST

Nicolas Boissel: reports personal fees from Sanofi. Alessandro Rambaldi: reports advisory board, personal fees and non‐financial support from Amgen, Celgene, Gilead, Italofarmaco, Jazz, Novartis, Pfizer and Roche. Giuseppe Rossi: reports personal fees from Sanofi. Andrey Sokolov: reports grants, personal fees and non‐financial support from Amgen, Novartis and Pfizer, grant and non‐financial support from Astellas. Ulla Wartiovaara‐Kautto: reports personal fees from Celgene, Pfizer and Sanofi. Giovanni Abbadessa, Alice Gosselin, Corina Oprea, Sandrine Macé: are employees of Sanofi and may hold shares and/or stock options in the company. Patrice Chevallier, Vadim Doronin, Laimonas Griskevicius, Alexey Maschan, James McCloskey, Xavier Thomas: have nothing to disclose.

## AUTHOR CONTRIBUTION

All authors were involved in the acquisition of data. Sanofi authors were involved in the analysis and interpretation of data. All authors were involved in the drafting and revision of the manuscript. All authors have given their approval for the manuscript to be published and are accountable for the accuracy and integrity of the results.

## ETHICAL APPROVAL STATEMENT

The protocol was approved by independent ethics committees and institutional review boards at all participating institutions. Written informed consent was obtained from all participants prior to inclusion in the study. The study was conducted in accordance with the Declaration of Helsinki and the International Conference on Harmonization Guidelines for Good Clinical Practice.

## Supporting information

Data S1

## Data Availability

Qualified researchers can request access to patient‐level data and related study documents including the clinical study report, study protocol with any amendments, blank case report forms, statistical analysis plan, and dataset specifications. Patient‐level data will be anonymized, and study documents will be redacted to protect the privacy of trial participants. Further details on Sanofi's data sharing criteria, eligible studies, and process for requesting access are at: https://www.clinicalstudydatarequest.com.
