# Supplementary material for: Isatuximab monotherapy in patients with refractory T‐acute lymphoblastic leukemia or T‐lymphoblastic lymphoma: Phase 2 study
Source: Cancer Med. 2022 Feb 2;11(5):1292–8. doi: 10.1002/cam4.4478 (PMC8894690; doi:10.1002/cam4.4478)
Supplement: Supplementary file 1 — Data S1 [file CAM4-11-1292-s001.pdf]

ISATUXIMAB MONOTHERAPY IN PATIENTS WITH RELAPSED/REFRACTORY T-ACUTE LYMPHOBLASTIC LEUKEMIA OR T-LYMPHOBLASTIC LYMPHOMA: PHASE 2 STUDY

Authors: Nicolas Boissel, Patrice Chevalier, Vadim Doronin, Laimonas Griskevicius, Alexey Maschan, James McCloskey, Alessandro Rambaldi, Giuseppe Rossi, Andrey Sokolov, Ulla Wartiovaara-Kautto, Corina Oprea, Giovanni Abbadessa, Alice Gosselin, Sandrine Macé, Xavier Thomas

Article URL: (TBC) | **Trial registration:** NCT02999633 | **@Nicolas Boissel** (TBC)

Study design and treatment

Inclusion criteria

- Relapsed ALL or relapsed AML
- Refractory to the most recent treatment
- >16 years at time of consent

→

Isa 20 mg/kg/week
Dexamethasone 10 mg/m²
ALL cohort: vincristine 1.5 mg/m²
ALL cohort: doxorubicin 10 mg/m²
ALL cohort: cyclophosphamide 440 mg/m²
AML cohort: cytarabine 60 mg/m²

P2 Phase **2**

Open-label

14 patients recruited

Summary of disease characteristics

Median age, years (range)
33.0 (16.0–74.0)

Initial diagnosis, *n* (%)

T-acute lymphoblastic leukemia	11 (78.6)
T-lymphoblastic lymphoma	3 (21.4)

At least one previous ASCT, *n* (%)
8 (57.1)

Number of prior lines of treatment, median (range)
5.5 (2.0–12.0)

Number of prior salvage therapy regimens, median (range)
Number of patients (*n* = 6)
2.5 (1.0–3.0)

Time from initial diagnosis to first drug administered, median years (range)
1.39 (0.4–6.8)

CD38 receptor occupancy

CD38 receptor density of cancer cells at baseline (sABC/cell)‡, median (range)

White blood cells (*n* = 10)	11,093.5 (181–76,131)
Bone marrow (*n* = 10)	6,817.5 (108–44,872)

CD38 positivity at baseline, median % (range)

White blood cells (*n* = 10)	87.50 (20.9–98.4)
Bone marrow (*n* = 10)	90.95 (12.2–98.6)

CD38 receptor occupancy of cancer cells at baseline, median % (range)

White blood cells (*n* = 10)	61.60 (24.0–84.8)
Bone marrow (*n* = 10)	52.31 (29.4–73.9)

CD38 receptor occupancy of cancer cells on treatment, median % (range)

White blood cells (*n* = 4)	57.96 (26.6–75.4)
Bone marrow (*n* = 3)	63.99 (61.7–73.0)

Overall response rate

ORR

Complete response (CR)
0

Complete with incomplete peripheral recovery (CRi)
0

Progressive disease, *n* (%)
12 (87.5)†

Responders, CR or CRi, *n* (95% CI)
0 (0.0%–23.2%)

Conclusion

T-ALL is an aggressive disease and the expression of CD38 is not sufficient to allow a biologic agent to be effective as monotherapy in heavily pretreated patients. Most patients in this study discontinued treatment before optimal blood levels of isatuximab could be reached. Despite the low efficacy of isatuximab in the current study, it is likely that the use of immunotherapy medication in T-ALL will be expanded through logically targeted approaches, together with advances in the design of T-cell therapy and clinical experience and will provide restorative options beyond chemotherapy and targeted treatments.

ALL, acute lymphoblastic leukemia; AML, acute myeloid leukemia; ASCT, autologous stem cell transplantation; CD, cluster of differentiation; CI, confidence interval; CR, complete response; CRi, complete response with incomplete peripheral recovery; Isa, isatuximab; kg, kilogram; mg, milligram; n, number; sABC, surface antibody binding capacity. †11 patients had progressive disease as best response (one patient, who withdrew from the study because of suspected progressive disease, had his post-treatment cancer therapy started 1 day before the confirmation of disease progression). ‡CD38 receptor density and receptor occupancy data at baseline were available for 10 out of 14 patients in the study.
